# Advances in Endothelial Cell Biology: From Knowledge to Control

**DOI:** 10.3390/ijms23126403

**Published:** 2022-06-08

**Authors:** Béatrice Charreau

**Affiliations:** CHU Nantes, Nantes Université, INSERM, Center for Research in Transplantation and Translational Immunology, UMR 1064, Institut de Transplantation, Urologie et Néphrologie (ITUN), F-44000 Nantes, France; beatrice.charreau@univ-nantes.fr

The aim of this Special Issue is to provide an overview of recent investigations in the field of endothelial cell (EC) biology that advance our understanding of the molecular mechanisms that trigger normal EC functions and dysfunctions in pathologies and to demonstrate how improved knowledge of EC biology may lead to the discovery of novel molecular diagnostic technologies and targeted therapeutics. The main messages from the contributions are summarized below.

## 1. Include EC Diversity to Improve Models and Design Targeted Therapeutics

The Special Issue reflects the need to take into account the heterogeneity of ECs among organs and tissues and to develop EC-targeted therapeutic strategies in experimental models and for clinical applications. Illustrating this point, this issue displays studies using a variety of ECs to explore dedicated functions including human umbilical vein ECs [1], pulmonary [2], renal glomerular [3], cerebral [4] and tumor ECs [5] but also adherent versus non adherent ECs [6] and ECs isolated from adults versus young children [3]. A better understanding of microvascular ECs’ heterogeneity will provide a roadmap for developing novel therapeutics to target the endothelium. In their review, Yang et al. discuss the morphological and functional variations in differently originated microvascular endothelia and how these variances affect systemic functioning in response to inflammation [7]. They also provide an overview of emerging in vivo and in vitro models and techniques, including microphysiological devices, proteomics, and RNA sequencing used to study the cellular and molecular heterogeneity of endothelia from different organs. Feitz et al. isolated human renal glomerular microvascular ECs from adults and from young children in an attempt to define why infection with Shiga-toxin-producing *Escherichia coli* bacteria causes the development of hemolytic uremic syndrome, a thrombotic microangiopathy with hemolytic anemia and thrombocytopenia affecting the kidneys, mostly in children under the age of 5 years [3].

## 2. Endothelial Cell Signaling in Endothelial Cell Function and Dysfunction

In the endothelium, the Rho family GTPases regulate junctional stabilization, cell motility and adhesion processes and play a crucial role in angiogenesis and endothelial barrier integrity. The Rho guanine nucleotide exchange factors (RhoGEFs) family contains around 70 members among them. Trio, which possess three enzymatic domains, has shown its importance in the endothelial cells’ morphology and barrier function. In their review, Kempers et al. focus on the function of Trio signaling in the healthy endothelium and on Trio dysregulation in vascular-related inflammatory diseases [8]. The migration of ECs is an essential process for the blood vessel formation. The activation of Rac1 GTPase mediates the migration of ECs by the actin cytoskeleton reorganization. Actin isoforms have tissue-specific functional features that depend on the cell type. In ECs, cytoplasmic β-actins and γ-actins are involved in the endothelial barrier function. By focusing on the role of actins in the functional activity of ECs, the contribution of cytoplasmic actins to angiogenesis and endothelial-to-mesenchymal transition (EndoMT), reported by Duginal et al. [9], are promising for further investigations, for fundamental cell biology, and for practice medicine. Tumors such as Glioblastomas, a subset of aggressive brain tumors, deploy several means to increase blood vessel supply dedicated to the tumor mass. Rosinska and Gavard review the specificities of each neovascularization modality in glioblastoma and how they can be hampered mechanistically in the perspective of anti-cancer therapies [5]. Zabroski et al. demonstrate that lipid rafts stabilize VEGFR2 and its associated signal transduction activities required for angiogenesis. These findings support the idea that the modulation of lipid rafts may provide a means to regulate the sensitivity of ECs to VEGF stimulation, a perspective to improves outcomes in the treatment of certain cancers [10]. EndoMT is a cell transdifferentiation process, where ECs progressively lose endothelial specific markers and functions, acquire a mesenchymal phenotype that results in increased motility, and loss of barrier function and cell junctions. In this Special Issue, Pham et al. show that the long non-coding RNA lncRNA AERRIE is expressed in ECs, is upregulated by EndMT, and demonstrates its role in the molecular cascade, resulting in ECs transitioning to mesenchymal cells [11].

Questioning the SARS-CoV-2 virus pathophysiology, data from Jana et al. reveal that the S1 spike glycoprotein alone can damage pulmonary ECs manifested by impaired mitochondrial function and increased glycolysis coupled with pro-inflammatory cytokine release, which, overall, can lead to endothelial activation and loss of barrier function [2]. The presence of hemoglobin during treatment with S1 protein as a mean to counterbalance hypoxic environment failed to inhibit S1 signaling in ECs. As a link between infection and inflammation, Lindkvist et al. investigate how the IL-6 family cytokines, which can be found in the circulatory system during physiological or pathological conditions, may use selective receptors and signaling pathways to influence endothelial function and response [1]. Investigating the pathophysiology of hypertension in hypertensive mice after the administration of angiotensin II, Kij et al. show that inhibiting thrombin prevents the development of endothelial dysfunction, increased systemic nitric oxide (NO) bioavailability, and limited endothelial inflammation but did not reduce elevated blood pressure and aortic thickening induced by angiotensin II [12]. To elucidate the regulation of p62-mediated autophagy under atherosclerotic conditions, Kim et al. investigated the inhibition of p62 and E3 ligases in ECs cocultured with macrophages under inflammatory stimulation by oxLDL [13]. The mechanism of autophagy in ECs was also explored using chloroquine, a drug known to inhibit autophagy flux by impairing autophagosome–lysosome fusion, which induces an accumulation of lysosomes and autophagosomes in ECs, suggesting that the lipid deposits were related to lysosomotropism and autophagy inhibition by chloroquine [14].

## 3. Endothelial Cells as Therapeutic Targets and Sources of Non-Invasive Biomarkers

The endothelium has also been proposed as a cellular target to evaluate the biological impact of urban particulate matter (UPM), small particles present in the air that contribute to inflammation and cardiovascular diseases, and allowed researchers to establish a direct association between UPM and the activation of the complement system in a cell culture model illustrating how air pollution may affect immunity [15]. In another way, experimental models using human EC cultures are being developed to optimize the endothelialization of biomaterial surfaces and for the in vitro assessment of cardiovascular implant material candidates [6]. 

Approaches to circumvent EC activation and dysfunction using treatment with cyclic nitroxides, namely, 4-methoxy tempol (4-MetT), showed significant inhibition or reversal of the inflammatory pattern mediated by Serum Amyloid A on cultured ECs, but this remains to be validated in vivo [16]. Polyphenols extracted from medicinal plants also provide interesting drugs to decrease inflammation and immunoregulation at the EC level [17]. Polyphenols display protective effects on inflammatory and permeability markers as well as monocyte recruitment on cerebral ECs exposed to hyperglycemic condition [4], indicating their relevance for pharmacological strategies aiming to limit cerebrovascular disorders in diabetes.

ECs possess a multilevel machinery to deliver messages to distant cells such as cells circulating in the blood vessel including platelets, leucocytes, other vascular cells, or normal and tumor cells in tissues. The secretory pathways, granules, extracellular vesicles, generating the endothelial secretome, and tunneling nanotubes (TNTs) used for intercellular communication between ECs and distant cells are also reviewed [18]. Efficient for adjusting thrombosis, angiogenesis, inflammation, infection, and immunity, these processes may also provide future therapeutic and diagnostic tools. As a consequence of the secretory activity, ECs may also provide a cell-specific source of non-invasive biomarkers. Miranda et al. reviewed the use of circulating cell free DNA (cfDNA) to explore EC dysfunction and cardiovascular diseases [19]. Although ubiquitous and not specific to the endothelium, the soluble form of RAGE is another potential biomarker of coronary artery disease reviewed by Kim et al. that may be used, most probably, in combination with other biomarkers to characterize vascular pathologies [20].
